# Hospital mortality predictive factors following Rapid Response Team activation

**DOI:** 10.1186/cc13272

**Published:** 2014-03-17

**Authors:** H Palomba, F Piza, M Jaures, A Capone

**Affiliations:** 1 Hospital Israelita Albert Einstein, Sao Paulo, Brazil

## Introduction

The Rapid Response Team (RRT) represents an important advance in the management of deteriorating ward patients and is recommended as a patient safety measure. Most studies on RRT evaluate the effects of its implementation on rate reduction of cardiopulmonary arrest outside the ICU and hospital mortality, with limited information on the criteria for RRT calls and predictive factors associated with hospital mortality [1],[2]. Therefore, our objective was to determine what factors are associated with hospital mortality for patients seen by the RRT at the Hospital Israelita Albert Einstein (HIAE).

## Methods

A total of 1,051 patients assessed by RRT between January and December 2012 at the HIAE, a general hospital with 650 beds, were included in this study. Multivariate analysis was used to evaluate what variables were associated with hospital mortality. Early RRT call was defined as RRT activation <48 hours from hospital admission and late RRT call if it happened >48 hours from hospital admission.

## Results

The mean age was 64 ± 19.8 years and 48% (*n *= 513) were male. There were 513 (48.9%) early calls and 537 (51.1%) late calls. The main reasons for RRT activation were respiratory failure in 18.2% (*n *= 191) and severe sepsis/septic shock in 13.1% (*n *= 138). The distribution of RRT activation was uniform over the 24-hour period, with 50.5% (*n *= 531) of calls during the day (7:00 a.m. through 7:00 p.m.) and 49.5% (*n *= 520) overnight (7:00 p.m. through 7:00 a.m.). A total of460 patients (43.7%) were admitted to the ICU. The multivariate analysis showed the following variables as significantly associated with hospital mortality: age (OR 1.03; 95% CI 1.01 to 1.04), late (>48 hours) RRT call (OR 2.73; 95% CI 1.79 to 4.71), acute change in oximetry saturation to <90% (OR 1.94; 95% CI 1.28 to 2.95) and acute change in respiratory rate to <8 or >28 breaths/minute (OR 1.79 95% CI 1.09 to 2.94).

## Conclusion

In this study, hospital mortality predictive factors for patients seen by the RRT were: age, acute respiratory failure and late RRT call.
